# The Quality of Tuberculosis Services in Health Care Centres in a Rural District in Uganda: The Providers' and Clients' Perspective

**DOI:** 10.1155/2014/685982

**Published:** 2014-09-07

**Authors:** Lilian Bulage, Juliet Sekandi, Omar Kigenyi, Ezekiel Mupere

**Affiliations:** ^1^Makerere University, College of Health Sciences, School of Public Health-Kampala, Uganda; ^2^National Tuberculosis and Leprosy Programme-Kampala, Uganda; ^3^National Tuberculosis Reference Laboratory-Kampala, Uganda; ^4^College of Public health, University of Georgia, 101 Buck Road, Athens, GA 30602, USA; ^5^Department of Paediatrics & Child Health College of Health Sciences, Makerere University-Kampala, Uganda

## Abstract

Quality of care plays an important role in the status of tuberculosis (TB) control, by influencing timely diagnosis, treatment adherence, and treatment completion. In this study, we aimed at establishing the quality of TB service care in Kamuli district health care centres using Donabedian structure, process, and outcomes model of health care. A cross-sectional study was conducted in 8 health care facilities, among 20 health care workers and 392 patients. Data was obtained using face-to-face interviews, an observation guide, a check list, and record review of the TB unit and laboratory registers. Data entry and analysis were done using EPI INFO 2008 and STATA 10 versions, respectively. A high number 150 (87.21%) of TB patients were not aware of all the signs to stop TB medication, and 100 (25.51%) patients received laboratory results after a period of 3–5 working days. The major challenges faced by health workers were poor attitude of fellow health workers, patients defaulting treatment, and fear of being infected with TB. One of the worst performance indicators was low percentage of cure. Comprehensive strengthening of the health system focusing on quality of support supervisions, patient follow up, promoting infection control measures, and increasing health staffing levels at health facilities is crucial.

## 1. Introduction

Tuberculosis (TB) remains a major public health problem worldwide. There were 8.6 million new TB cases and 1.3 million TB deaths in 2012 [1]. The African region alone accounted for 27% of the world's cases and the highest rates of cases and deaths relative to population (225 incident cases per 100,000 on average and more than double the global average of 122) [1].

Uganda's TB burden remains unacceptably high, ranking 19th among the 22 high burden TB countries globally. In 2010, Uganda only reached 57% case detection rate (CDR) as opposed to the WHO target of 70%. Kamuli, a rural district located in the South Eastern TB Zone as designated by the Uganda National TB and Leprosy Program (NTLP), had 35.7% CDR for new smear positive cases. Out of the 225 sputum smear positive cases captured in 2010, 84 (37.3%) got cured, 106 (47.1%) completed treatment, 13 (6%) died, 1 (0.4%) was a failure, 16 (7.1%) defaulted treatment, and the other 5 (2.2%) were transferred to another district (Kamuli Annual Report on TB/Leprosy Control 2011).

Literature suggests that poor quality of care may result from discrepancies in documentation such as underreporting and gaps in the continuum of care services that the patients receive [1]. Donabedian developed the structures, process, and outcomes model as a framework for assessing the quality of health care services [2]. Structure consists of physical health facility, medical equipment, and staff characteristics. Processes of care involve interactions between users and the health care structures, the actual delivery, and receipt of care. Outcomes are consequences of care. Structures as well as processes may influence outcome, indirectly or directly [3]. There is low comprehensive knowledge about TB in the general population including lack of information on the TB service delivery points among other factors [4].

In Uganda, TB health care services at the district level are delivered through a continuum of various health facility structures designated as hospitals and Health Centers IV and III. Depending on the structural level of the health facility, the processes of care may begin with patient screening by health providers using specific case screening tools like the desk aids, intensified TB case finding forms (ICF), and the suspect register. The screening process could happen at outpatient clinics, specialized HIV/AIDS clinics, waiting rooms, and/or in-patient wards. The TB suspects are then registered and referred to the laboratory where bacteriological diagnosis is done on two samples (one spot and one early morning) by either Ziehl Neelsen (ZN) or fluorescence microscopy and documentation is done in the laboratory TB register [5]. At all the TB service points, the TB suspects or patients are supposed to be given specific information about TB symptoms, diagnosis, treatment, and follow up. Confirmed TB patients are registered and information is transferred to the health unit TB register. Patients are started on antituberculosis treatment, monitored monthly while on treatment for clinical and bacteriologic response, and evaluated at the end of treatment to document the relevant outcomes.

**Figure 1:**
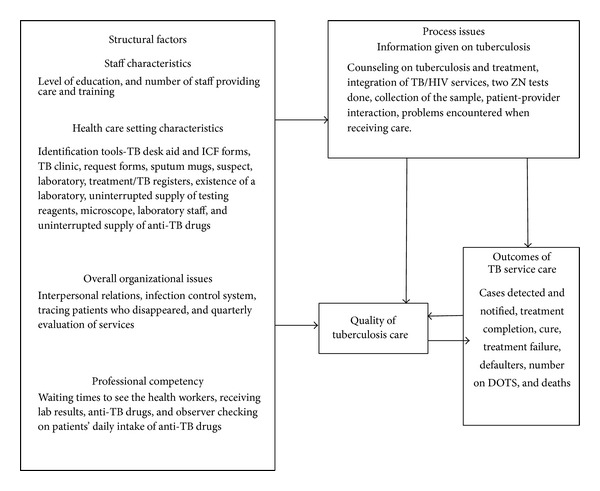
Conceptual frame work for quality of TB service in Kamuli district: adopted from the Donabedian model of quality of health care.

Given the various points within the health services that have to be traversed, the services patients receive can be compromised at the structural process or outcome levels leading to poor quality of TB care. However, the contributing structural and process factors have not been systematically studied and documented. Therefore, this study sought to establish the factors that influence quality of services among new smear positive TB cases in health care centers in Kamuli district. We adapted the Donabedian's structure, process, and outcomes model as the conceptual framework to study the factors that influence quality of TB services (Figure 1).

The results can inform the NTLP about the status of the quality of TB services in this rural setting and highlight pragmatic ways in which services can be improved.

## 2. Methods

### 2.1. Study Design

A cross-sectional study was conducted in Kamuli district using mixed methods. Both qualitative and quantitative data were collected. The patients' overall perception of the services together with the WHO, NTLP, and International Standards for Tuberculosis Care (ISTC) were used to judge the overall quality of services offered at the facilities. Quality was measured by the difference between expected and actual performance to identify opportunities for improvement.

### 2.2. Study Area

Kamuli district located in the South Eastern TB Zone of Uganda, 150 kms from Kampala, was the study area selected on the basis of having poor TB outcomes for the new sputum smear positive cases in the year 2010. The district has 2 hospitals, 2 health center fours (HCIV), and 11 health centre threes (HCIII). Malaria, acute respiratory infections, intestinal worms, sexually transmitted infections (STI), and HIV and AIDS are the commonest causes of morbidity in the district.

### 2.3. Study Population

The study was carried out at tuberculosis diagnostic and treatment health units and the study participants were health unit in-charges, in-charges antiretroviral therapy (ART) clinics, TB focal persons, TB suspects, and confirmed TB patients aged 15 years and above and found at the health facilities on the day of visit. The above health workers were selected based on the knowledge base they had on TB services and logistics for TB care.

### 2.4. Inclusion and Exclusion Criteria

Diagnostic treatment facilities were included in the study. All the health workers (HW) who participated had worked at the facility for at least 3 and above months. Any TB suspects aged 15 years and above who had gone through all the service points up to the level of getting results from the laboratory, confirmed TB cases who had gone up to the point of receiving treatment or were already on treatment and found at the health facility at the time of the visit were included in the study. Patients too weak to be interviewed as judged by the research assistant were excluded and transfer ins from the health facility treatment register were also excluded from both the record review and interviews.

### 2.5. Sample Size

To establish the quality of the structural and processes of TB service care, 20 health workers and 392 (determined using the Leslie Kish formula) patients were interviewed after accounting for a 10% nonresponse rate. The number of patients to be interviewed at each facility was determined using the probability proportionate to size method. Kamuli Government hospital 182, Kamuli Mission Hospital 69, Namwendwa HCIV 53, Bulopa HCIII 10, Balawoli HCIII 32, Butansi 16 HCIII, and Bupadhengo HCIII had 30 patients interviewed. There were no patient interviews in Nankandulo HCIV, because at that time laboratory TB diagnosis had been stopped though we went ahead to interview the health workers. To establish the level of TB service care outcomes, all the study facilities were included and data for the new smear positive patients captured in 2010 was reviewed.

### 2.6. Sampling Procedure

A list of all the diagnostic treatment units (DTU) was obtained from the district health officer (DHO) to ensure that a representative number was considered for the study. From the list, the 2 hospitals (Kamuli Government Hospital and Kamuli Mission Hospital), 2 HCIV (Nankandulo HCIV and Namwendwa HCIV) were considered. However, since the HCIIIs were many, 2 high work load (Balawoli HCIII and Bupadhengo HCIII) and 2 low work load health facilities (Butansi HCIII and Bulopa HCIII) were considered for the study. Selection of health workers was done purposively. Tuberculosis patients (suspects or TB cases) exiting were enrolled to participate in the study consecutively until the required sample size was got. Data recorded in the TB laboratory and unit TB register for the year 2010 were reviewed to collect information on the number of ZN smears, cases notified, treatment completion, cure, defaulting, and death.

### 2.7. Data Collection

Information on quality of the structural and process factors was got by use of an on-site observation guide, a checklist, and holding face to face interviews using a semistructured questionnaire with in-charges of health care units, in-charges ART clinics, and TB focal persons. Tuberculosis suspects and cases were too face-to-face interviewed on the quality of process, some structural aspects, and overall rating of the facility in terms of quality. Data in the TB laboratory register was also reviewed to get information on the number of suspects assessed and how many of the ZN tests had been done for each suspect for the year 2010. Information on outcomes of TB care was got by reviewing records in the health unit treatment TB registers for the year 2010 since it was expected that all the patients who were detected and started on treatment in that year had already had an outcome. To judge the overall quality of a particular health facility, the information collected about the quality of structural, process, and level of outcomes of TB service care was summarized into an indicator table which eventually led to a final judgment of the quality of services offered. Least performance score implied good quality services while the greatest score implied poor quality services offered by a facility (Table 1). The best and worst quality performance facilities and indicators were identified.

### 2.8. Quality Control

Two research assistants with knowledge on TB care were recruited for each facility and trained for 2 days on issues concerning this study. The research assistants were both English and Lusoga speakers whose least level of education was ordinary level.

### 2.9. Data Management

Data was entered and checked using EPI INFO 2008 version and exported to STATA version 10 for analysis. Missing data was ignored since it was small compared to the total data set count. Univariate analysis was run to obtain the baseline characteristics of the patients. Univariate analysis was also done to establish proportions, frequencies, and percentages regarding the performance of the health units on the different parameters of TB service delivery. For confidentiality reasons, the health facility names were replaced with letters when reporting on the number of ZN tests done, the TB outcomes, and the general quality performance of each facility. In order to clearly indicate the most serious and common problems faced by health care workers, all the problems mentioned by each of them were summarized and key issues were stated. For each problem, the number of times it was mentioned by the different health care workers was tallied and the total was recorded.

### 2.10. Ethical Considerations

Approval was got from the Uganda National Council of Science and Technology, through Makerere University, School of Public Health, and permission was sought from the district health officer. Informed consent was also sought from the respondents of 18+ years and care takers/parents of patients 15 to 17 years of age. Assent was sought from respondents of 15 to 17 years of age.

## 3. Results

### 3.1. Baseline Characteristics of the Patients Attending Health Facilities in Kamuli District, March–May 2012

The study consisted of 392 respondents of which 220 (56.12%) were suspects, 33 (8.42%) were newly confirmed TB cases, and 139 (35.46%) were TB cases on treatment. Most of the respondents 226 (58.80%) were female, belonging to age category 25–34, 123 (31.38%), and 35–44, 128 (32.65%), 294 (75%) were rural residents, and 249 (63.52%) were peasant farmers who had attained 191 (48.72%) primary education as the highest level of education reached and the biggest numbers of the respondents were protestants (Table 2).

### 3.2. Quality of the Structural Aspects of TB Service Care

#### 3.2.1. Structural and Overall Organizational Characteristics among the Health Facilities

All the 8 facilities had suspect, laboratory, and unit registers, intensified case finding forms, a full set of TB testing reagents, a microscope, anti-TB drugs, and a system of tracing patients who either tested positive or started on treatment and disappeared. All the 8 facilities were giving return appointments to patients who were smear negative and had been put on antibiotics at the time of study. Only 3/8 facilities had the TB management desk aid, only 2/8 were using and filling in the TB suspect registers consistently, and 7/8 had sputum mugs; all the 8 facilities had no evidence of their microscopes having been serviced in the last one year, 1/8; that is, Nankandulo HCIV, did not have a system of identifying coughing patients from waiting areas, wards, and so on (Table 3).

### 3.3. Laboratory Staffing Levels of the Health Facilities

**Figure 2:**
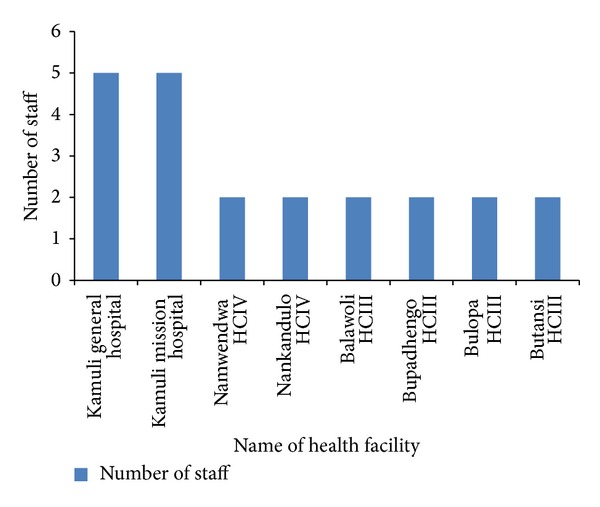
Laboratory staffing levels of health facilities in Kamuli district, March–May 2012.

Each of the 2 HCIVs and the 4 HCIIIs had 2 laboratory personnel. Each of the hospitals only had 5 laboratory personnel (Figure 2). The most qualified laboratory staffs were technicians with a diploma in medical laboratory technology while the least qualified were microscopists who are basically on-job learners and the entomological assistants who were formerly working with the sleeping sickness control program.

### 3.4. General Perception of Interviewed Staff on Staffing Levels and Supervision among the Health Facilities

All the 20 health workers interviewed perceived the number of staff not enough to cover the TB work load and the frequency of supervision was said to be on a monthly basis by the majority of health workers (17/20).

### 3.5. Quality of the Processes of TB Service Care

#### 3.5.1. Patient Reported Process Characteristics of TB Care at the Health Facilities

Forty-nine (12.53%) and 40 (10.23%) of the patients got to know about the existence of TB services from friends/relatives/someone who had ever used the services and from the media, respectively; 52 (13.27%) patients were not informed that they would transmit TB to others, 94 (23.98%) were not informed how to stop spreading TB to others, 55 (31.98%) patients on treatment and newly confirmed cases were not informed of the side effects of the TB drugs, 150 (87.21%) of patients who had been started on treatment and the newly confirmed were not aware of all the signs to stop TB medication, 100 (25.51%) received laboratory results after a period of 3–5 working days and 57 (33.14%) patients who tested positive for TB received their drugs after a period of 2 to 5 working days (Table 4).

### 3.6. Number of ZN Tests Performed by Laboratories for Suspects Captured in the Year 2010

Out of the total 1453 suspects identified in the year 2010, we had 1384 (95.3%) microscopically being assessed by the health facilities. Out of the 1384, we had 1115 (76.7%) suspects having had all the two recommended smears done. However, 271 (18.5%) suspects had only one smear done. Out of the 1453 suspects, 69 (4.7%) who were supposed to have had microscopy done on their sputum did not have it done at all (Table 5).

### 3.7. Challenges Which Need to Be Improved upon as Reported by Patients and Health Care Providers

**Figure 3:**
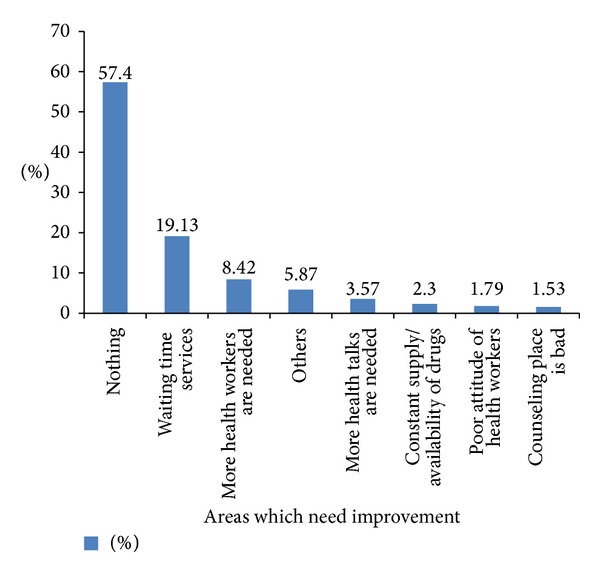
Challenges which need to be improved upon as reported by patients in Kamuli district health facilities, March–May 2012. Note: others included: need for food, wheel chairs, out-patient department needs improvement, improve on the infrastructure, and reduce the quantity of drugs.

Waiting times to receive services (19.13%), need for more workers (8.42%), and health talks (3.57%) are some of the key issues patients felt should be improved upon (Figure 3). The major challenges faced by the health care workers as reported by themselves were poor attitude of some health workers (mentioned 8 times), patients who default (mentioned 7 times), and fear of being infected with TB (mentioned 6 times). The other issues which providers felt should be improved upon were understaffing (mentioned 5 times), patients who do not keep appointment (mentioned 5 times), long waiting time to receive laboratory results, inadequate knowledge on TB, limited space for TB work, and fear of the CB-DOTS program not working as expected.

*Note*. Frequency of challenges faced by health workers is equivalent to the number of times the challenge was mentioned by the health workers.

### 3.8. The Levels of TB Service Care Outcomes for New Sputum Smear Positive Cases for the Year 2010

The 8 study facilities contributed 176 new smear positive TB patients to the total number detected by the district in 2010. Out of the 176, 71 (40.3%) patients completed their treatment, 69 (39.2%) were declared cured by microscopy, 1 (0.6%) was a treatment failure, 22 (12.5%) defaulted treatment, and 12 (6.8%) died (Table 6).

### 3.9. General Quality Performance of Kamuli District Health Facilities as Judged against Guidelines/Indicators

Facility H (Hospital) and G (Hospital) were the worst performing facilities followed by D (HCIII). The best performance facilities were C (HCIII) and B (HCIII) (Table 7).

Though we did not have data on patients' views for F (HCIV) to allow scoring of certain quality parameters like waiting time to receive laboratory results, anti-TB drugs, patients awareness on adverse effects of drugs, and advise to test for HIV, we still went ahead to score the rest of the parameters whose data was available and finally concluded on the facilities performance. For the few parameters assessed, the score was already high, so we concluded that the facility's quality of TB service care was poor.

The worst performance indicators were making sure that every suspect is tested for TB with at least one ZN test. That was followed by ensuring two ZN tests for every suspect and the number cured whose percentage was very low (Table 7).

*Note.* For more information on how the scores were generated and what they meant, check Table 1: performance indicators/guidelines against which quality for Kamuli district health facilities was judged.

The patients overall perception of the services together with the WHO, NTLP, International standards for tuberculosis care (ISTC), and the researchers' own set perception about the quality of services offered were used to judge the overall quality of services offered at the facilities/level of execution of the different indicators.

## 4. Discussion

In this cross-sectional study conducted to assess quality of TB health services in a rural district, we found that the patients were not receiving adequate information about TB from the health providers. This is evidenced by a number of patients who were not informed about the possibility of transmitting TB to others, how to stop transmitting TB, the side effects of TB drugs, and signs of side effects in order to stop TB medications. Limited information among the patients could explain the poor outcomes such as the high default and deaths rates reported in the study. Health care providers often try to supply information to patients and to motivate them and recognize the importance of behavioral skills in improving health. However, there is evidence that, in practice, health providers are constrained by time and hence they give limited information [6]. In order to solve the problem of inadequate information, the TB program could develop a standardized approach to patient health education where the health providers use structured guidelines and also provide user-friendly translated information, education, and communication (IEC) materials including visual aids to all TB patients. A systematic review showed that overall education or counseling interventions may increase successful treatment completion although the magnitude of benefit is likely to vary depending on the nature of the intervention and the setting [7].

Patient waiting time to receive services is one of the major challenges hindering TB control as evidenced by a number of patients who received laboratory results after a period ranging from three to more than five working days. The National TB Reference Laboratory standard operating Procedure states that microscopy results should be reported within 24 hours; anything beyond 24 hours is considered unacceptable.

According to the ministry of health guidelines, a patient is supposed to be started on treatment as soon as they are identified to have TB; yet the findings reveal a number of patients taking days without treatment initiation. Gulu Regional Referral Hospital project also revealed that over 50% of patients at Gulu TB Ward clinic in 2008 had a waiting time of 7 days from TB suspicion to treatment initiation [8]. Another study carried out in Botswana, on completeness and timeliness of treatment initiation after laboratory diagnosis of tuberculosis, showed that 11.8% patients had a delay in treatment initiation [9]. In Uganda and much of Africa, the TB patient load is increasing due to the underlying HIV epidemic that partly fuels the TB epidemic. Moreover, there is a shortage of skilled healthcare personnel to deliver health services leading to an overstretched health system [10, 11]. In order to improve the workflow and mitigate the waiting problems innovative ways of delivery of care such as task-shifting through training a cadre of lay health workers to perform simple tasks like collecting sputum and initial patient registration could be explored. Service care programs for HIV/AIDS have employed similar low-cost models of task-shifting and they have helped to relieve the strain on the health system [12, 13].

Adherence to recommended guidelines for smear microscopy was very low indicating poor performance almost across all health facilities. The study reveals 18.5%, and 4.7% of the TB suspects not having had a second smear and not having microscopy done at all instead of the two tests recommended by the guidelines. This highlights an important gap in the processes that could be leading to poor quality of service due to improper management of the patients and may perhaps imply lack of competence on the providers' side. Similarly, a study carried out in Botswana, on completeness and timeliness of treatment initiation after laboratory diagnosis of tuberculosis, also revealed that 10.2% patients had only one smear done [9].

Most of the TB patients on treatment had a treatment observer checking and ensuring on their daily intake of drugs, the NTLP recommends every patient to have a treatment observer. According to one of the health care workers in facility E (HCIV), the health care workers are not sure whether what they refer to as DOTS is actually working and they are not sure of whether the treatment observers are carrying out their role. Verification of such a finding using a community based study is needed for the government to be sure whether the DOTS programme is working as expected and yielding positive results. This doubt was also cited by a paper from Ethiopia, which recommended for an expanded community-based study to better guide quality DOTS programs. The number of patients declared cured is still wanting as seen from the outcome table; this actually means that patients just take medication but the issue of follow ups recommended at 2, 5, and 8 months of treatment is not being ensured by health facilities. The flow of patients and the location of the unit TB register need to be checked and organized so that no patient gets treatment without a follow-up test.

The study revealed a high proportion of treatment defaulters. This could be explained by the patients not getting adequate information about TB and a failing DOTS program. According to a qualitative study carried out in Norway on barriers and facilitators of adherence to TB treatment in patients on concomitant TB and HIV treatment, some of the barriers to treatment adherence were experiencing side effects, economic constraints, lack of food, and lack of adequate communication with health professionals among other factors [14].

All the health facilities had the basic essential requirements for TB diagnosis and treatment. This could be attributed to consistent monthly and quarterly supervisions by the district team, and a few times by the ministry of health officials as recommended by the ministry of health and presence of development and implementing partners like global fund, STAR-EC, and Plan-Uganda could also be reason for the essential requirements being in place. A study carried out on quality of tuberculosis care in Ethiopia also revealed delivery of materials, drugs, and supplies for tuberculosis control activities being fairly good [15].

All the facilities had the TB suspect register but only two were consistently using and filling the register. According to the guidelines, all the TB suspects identified in the health facility are supposed to be registered in the TB suspect register and are then sent to the laboratory for diagnosis. This could be attributed to health workers not knowing the purpose of such a register. According to the NTLP-Uganda, the suspect register provides a means of recording all symptomatic patients classified as suspects; it includes all patients who have a cough for two weeks or more. The suspect register is a useful tool to monitor sputa sent to the laboratory, for evaluating case detection and determining the prevalence of TB suspects at facility level. It is also useful for estimating supply levels needed for bacteriologic exams. The National TB and Leprosy Programme should think of evaluating the usage and filling of the suspect register in other districts before coming up with a conclusive way forward.

Our study points out to poor attitude of fellow health workers and fear of being infected with TB as some of the key challenges faced by health workers. Similarly, a cross-sectional study carried out in Ghana also pointed out that attitudes of healthcare workers towards TB patients and health staff's own fear of TB were some of the major problems hindering TB work and accelerating stigma [16]. The programme should place emphasis on participatory in-service training for health workers focusing on patient-centered continuous quality improvement, teamwork and, implementation of infection control practices. A similar approach to quality improvement was used in a TB program in Cape Town, South Africa [17].

The main limitations in our study were patients' exit interviews using a cross-sectional design; this has inherent potential for several biases such as social desirability bias and selection bias. The selection bias was minimized by sampling from different levels of health facilities and the social desirability bias was reduced by using volunteers who are not health workers to conduct the patient interviews. Since some of the results are from secondary TB program data that was not collected primarily for research, the potential for some inaccuracy cannot be ruled out. However, that was minimized by verification of data using the district TB registers, consulting the district TB leprosy focal person, and the various health facility staff met. Gaps in data due to missing information on some indicators and difficulty in accessing some registers could have compromised the quality of the record review results. Recalling accurately the presence of some items like reagents and drugs in the last one year may have compromised the study findings, but that was minimized by use of stock cards to verify the responses given. Despite the highlighted limitations, the study's greatest strength was the use of mixed methods, quantitative and qualitative methods of data collection to examine the complex aspects of quality of TB services and to provide some initial evidence that can be used to improve patient care.

## 5. Conclusion

The basic structural essential requirements for TB diagnosis and treatment were in place. However, the patients were not receiving adequate information about TB from the health workers, long patient waiting time to receive services at each of the levels of care, poor attitude of fellow health workers and health workers' own fear of being infected with TB, and the low percentage of cure are some of the key process issues hindering TB control in the rural setting. Comprehensive strengthening of the health system focusing on quality of support supervisions, patient follow up and promoting infection control measures and increasing health staffing levels at the health facilities are crucial.

## Figures and Tables

**Table 1:** Performance indicators/guidelines against which quality for Kamuli district health facilities was judged.

Health facility expected performance indicators or guidelines	Actual performance level	Source of data	Performance score
Structural performance guidelines/indicators			
All health workers participate in identification of TB suspects	1 = Yes 2 = No	Interviewed health facility heads, TB focal persons, and in-charges ART	1 = good 2 = poor
Waiting times for; (i) Receiving laboratory results (ii) Receiving treatment	(i) 1 = 24 hours 2 => 24 hours (50% and above of clients getting results after 24 hours) (ii) 1 = immediate 2 => 1 day	Interviewed patients	1 = good 2 = poor

Process performance guidelines/indicators			
Two sputum samples should be collected for diagnosis	All suspects should have two ZN smears done	Reviewed laboratory register data for 2010	1 = 81–100% = good 2 = 70–80% = fair 3 =< 70% = poor
The health facility in conjunction with the sub-county health worker, and community volunteers should trace all clients who have disappeared without getting treatment, and any other category of interest	1 = Yes 2 = No	Interviewed health facility heads, in-charges ART, and TB focal persons	1 = good 2 = poor
All TB suspects should be assessed using a symptom based approach	Percentage of TB suspects who were assessed by the laboratory with at least one ZN test	Reviewed the laboratory register and identified patients who were registered but no test done for them at all	1 = 0 patients = very good 2 = 1–3 patients = good 3 = 4 & > patients = poor
To assess and foster adherence to treatment, a patient-centered approach to administration of drug treatment, based on the patient's needs and mutual respect between the patient and the provider, should be developed for all patients. Supervision and support should be individualized and should draw on the full range of recommended interventions and available support services, including patient counseling and education	Each TB positive patients should be on community based-DOTs	Reviewed the unit TB treatment register	1 = all patients = good 2 = some patients not on DOTs = poor

Process performance guidelines/indicators			
HIV testing and counseling should be recommended to all patients with, or suspected of having, tuberculosis	Every TB suspect should be advised to test for HIV	Interview with patients	1 = all patients = good 2 = some patients not advised = poor
Each healthcare facility caring for patients who have, or are suspected of having, infectious tuberculosis should develop and implement an appropriate tuberculosis infection control plan	Presence of a system of identifying coughing patients from waiting areas and other places	Interviewed health facility heads, in-charges ART, and TB focal persons	1 = good 2 = poor

Outcome performance guidelines/indicators			
Cured	Percentage declared cured	Reviewed data in the treatment register for 2010	1 = 85–100% = good 2 = less than 85% = poor
Treatment failures	Number declared as failures	Reviewed data in the treatment register for 2010	1 = 0 patients = good 2 = 1 or > patients = poor
Defaulters	Percentage defaulters, no patient should default	Reviewed data in the treatment registers for 2010	1 = 0 patients = good 2 = 1–7 patients = fair 3 = 8 and > patients = poor
Died	Percentage died, no patient is expected to die	Reviewed data in the treatment register for 2010	1 = 0 = good 2 = 1–5 patients = fair 3 = 5 and > patients = poor

**Table 2:** Baseline characteristics of the patients attending health facilities in Kamuli district, March–May 2012.

Characteristic	Frequency (*N* = 392)	Percentage
Sex		
Male	165	42.20
Female	226	58.80
Type of patient		
Suspects	220	56.12
Newly confirmed TB cases	33	8.42
TB cases on treatment	139	35.46
Age category		
15–24	62	15.82
25–34	123	31.38
35–44	128	32.65
45–80	79	20.15
Residence		
Rural	294	75.0
Urban	98	25.0
Occupation		
Peasant farmer	249	63.52
Civil servant	26	6.63
Business	81	20.66
Students	27	6.89
Others^1^	9	2.30
Highest education attained		
None	69	17.60
Primary	191	48.72
Secondary	108	27.55
Tertiary	24	6.12
Religion		
Catholic	129	32.91
Protestant	170	43.37
Others^2^	93	23.72

Mean age of respondents = 35.87 SD = 11.63 min and max age was 15 and 80.

^1^Housewife, cattle keeper, and driver, ^2^Orthodox, “born agains”, seventh day Adventists (SDA), and Muslims.

**Table 3:** Structural and overall organizational characteristics among health facilities in Kamuli district, March–May 2012.

Characteristic	Frequency (*n* = 8)	Percentage
TB desk aid		
Available	3∗∗∗∗∗	37.5
Not available	5	62.5
TB suspect register-used consistently		
Yes	2∗∗∗∗	25.0
No	6	75.0
TB request forms		
Available	6	75.0
Not available	2∗∗∗	25.0
Sputum mugs		
Available	7	87.5
Not available	1∗	12.5
Evidence of service of microscope		
Available	0	0.0
Not available	8	100
System of identifying coughing patients		
No	1∗∗	12.5
Yes	7	87.5

*Bupadhengo HCIII, ∗∗Nankandulo HCIV, ∗∗∗Bulopa HCIII and Bupadhengo HCIII, ∗∗∗∗Bupadhengo HCIII and Butansi HCIII, ∗∗∗∗∗Balawoli HCIII, Kamuli General Hospital, and Kamuli Mission Hospital.

**Table 4:** Patient reported process characteristics of TB care at the health facilities in Kamuli district, March–May 2012.

Process characteristic	Frequency (*N* = 392)	Percentage
Source of information about TB services		
Referred by a health worker	294	75.19
Recommended by somebody who has ever used	49	12.53
From the media	40	10.23
Others	8	2.05
Informed that you would transmit TB to others		
No	52	13.27
Yes	340	86.73
Informed when you stop spreading TB to others		
No	94	23.98
Yes	298	76.02
Informed when next to come back for TB services		
No	64	16.33
Yes	328	83.67
Informed that TB is cured		
No	24	6.14
Yes	367	93.86
Informed about side effects of TB drugs		
No	55	31.98
Yes	117	68.02
Aware of all the signs to stop TB medication		
No	150	87.21
Yes	22	12.79
Informed about sputum follow-up tests at given points		
No	12	6.98
Yes	160	93.02
Informed about the link between HIV and TB		
No	24	6.12
Yes	368	93.88
Advised to take an HIV test		
No	25	6.38
Yes	367	93.62
HW explained to you how to collect the sample		
No	63	16.11
Yes	328	83.89
HWs explained things in a way you understand		
No	16	4.08
Yes	376	95.92
You received all the necessary information you need to know		
No	54	13.78
Yes	338	86.22
Had enough time to discuss problems with HWs		
No	110	28.06
Yes	282	71.94
Opinion about attitude of staff at the health facility		
Very good	127	32.48
Good	179	45.78
Fair	84	21.48
Poor	1	0.26
Time spent to receive lab results after handing in second sample		
0–2 working days	270	68.88
3–5 working days	100	25.51
More than five working days	22	5.61
Treatment observer checking on your daily intake of drugs		
No	23	16.55
Yes	116	83.45
Waiting time to see the health care worker		
Less than 1 hour	162	41.33
1 hour to 2 hours	188	47.96
More than two hours	42	10.71
Waiting time to receive anti-TB drugs		
0-1 day	115	66.86
2 working days	39	22.67
3 working days	12	6.98
5 working days	6	3.49

Note: Only the newly confirmed TB cases and TB cases on treatment were asked whether they had been told about the side effects of TB drugs, and whether they knew all the signs to stop TB medication (severe skin itching, change of eye colour, impaired vision, and severe vomiting), and whether they had been informed about follow up tests of TB at different points during the course of treatment (*n* = 172).

Note: Only the TB cases on treatment were asked whether they had a treatment observer checking on their daily intake of anti-TB drugs (*n* = 139).

Note: Only the newly confirmed TB cases and TB cases on treatment were asked how long they waited to receive anti-TB drugs (*n* = 172).

Note: Waiting time to receive anti-TB drugs was considered from the time of receipt of laboratory results.

Note: Waiting time to see the health care workers was considered from the time a patient reached the facility to seeing the clinician.

**Table 5:** Number of ZN tests performed by laboratories for suspects captured in Kamuli district health facilities for the year 2010.

Name health facility	Total suspects	Total suspects assessed by the lab	Total suspects with 2 smears done	Total suspects with 1 smear done	Suspects with no smear done at all
A (HCIII)	20	17	13	4	3
B (HCIII)	44	44	43	1	0
C (HCIII)	68	64	55	9	4
D (HCIII)	94	93	80	13	1
E (HCIV)	202	201	167	34	1
F (HCIV)	48	42	38	4	6
G (Hosp)	354	351	289	62	3
H (Hosp)	623	572	430	142	51

Total (%)	1453	1384 (95.3)	1115 (76.7)	269 (18.5)	69 (4.7)

**Table 6:** Outcomes of TB service care for new sputum smear positive cases in Kamuli district health facilities for the year 2010.

Name of health facility	Number of smear positives identified in 2010	Number started on treatment	Number on F-DOTS	Number on CB-DOTS	Completed treatment	Cured	Treatment failures	Defaulted treatment	Died
A (HCIII)	01	01	—	—	01	—	—	—	—
B (HCIII)	03	03	—	03	01	02	—	—	—
C (HCIII)	08	08	—	08	03	04	—	01	—
D (HCIII)	13	13	—	11	09	03	—	—	01
E (HCIV)	18	18	01	17	08	06	—	02	02
F (HCIV)	02	02	—	01	02	—	—	—	—
G (Hospital)	72	72	19	21	28	030	—	11	03
H (Hospital)	59	59	—	09	19	024	01	08	06

Total (%)	176	176	20 (11.4)	70 (40)	71 (40.3)	69 (39.2)	1 (0.6)	22 (12.5)	12 (6.8)

F-DOTS-facility-directly observed therapy-short course, CB-DOTS-community based-directly observed therapy-short course.

**Table 7:** General quality performance of Kamuli district health facilities as judged against guidelines/indicators.

Health facility	A	B	C	D	E	F	G	H	I	J	K	L	M	N	O	Total score
H (Hospital)	2	1	2	2	1	1	3	2	2	2	1	2	2	3	3	**28**
G (Hospital)	2	1	2	2	1	1	2	2	3	1	1	2	1	3	2	**26**
F (HCIV)	2	—	—	2	2	2	3	2	—	—	2	2	1	1	1	**20**
E (HCIV)	1	1	1	2	1	1	2	1	3	1	1	2	1	2	2	**22**
D (HCIII)	2	2	1	2	1	1	3	2	1	1	1	2	1	2	2	**24**
C (HCIII)	2	1	1	2	1	1	2	1	2	1	1	2	1	1	1	**20**
B (HCIII)	2	1	1	2	1	1	1	1	3	1	1	2	1	1	1	**20**
A (HCIII)	2	1	1	2	1	1	2	2	2	1	1	2	1	1	1	**21**

**Total score**	**15**	**8**	**9**	**16**	**9**	**9**	**18**	**13**	**16**	**8**	**9**	**16**	**9**	**14**	**13**	

Note: for both the health facilities and guidelines, least performance score implied good quality services while the greatest score implied poor quality services offered by a facility.

Key:

a-every staff participates on identification of tuberculosis cases, B-waiting to receive laboratory result, C-waiting time to receive treatment, D-two ZN tests done for every suspect, E-system of identifying coughing clients from waiting, areas, and other places, F-system of tracing lost clients, G-assessing every patient with at least one test, H-is every TB client on DOTs? I-TB patients aware of adverse effects/side effects of TB drugs, J-every suspect advised to test, for HIV, K-cases notified, L-number of patients cured, M-number of treatment failures, N-number of patients who defaulted, O-number of patients who died.
